# Supplementary material for: Prc1-rich kinetochores are required for error-free acentrosomal spindle bipolarization during meiosis I in mouse oocytes
Source: Nat Commun. 2020 May 27;11:2652. doi: 10.1038/s41467-020-16488-y (PMC7253481; doi:10.1038/s41467-020-16488-y)
Supplement: Supplementary file 1 — Supplementary Information [file 41467_2020_16488_MOESM1_ESM.pdf]

Supplementary information for “Prc1-rich kinetochores are required for errorfree acentrosomal spindle bipolarization during meiosis I in mouse oocytes”

Yoshida S., Nishiyama S., Lister L., Hashimoto S., Mishina T., Courtois A., Kyogoku H., Abe T., Shiraishi A., Choudhary M., Nakaoka Y., Herbert M. and Kitajima T.S.

Supplementary Figure 1

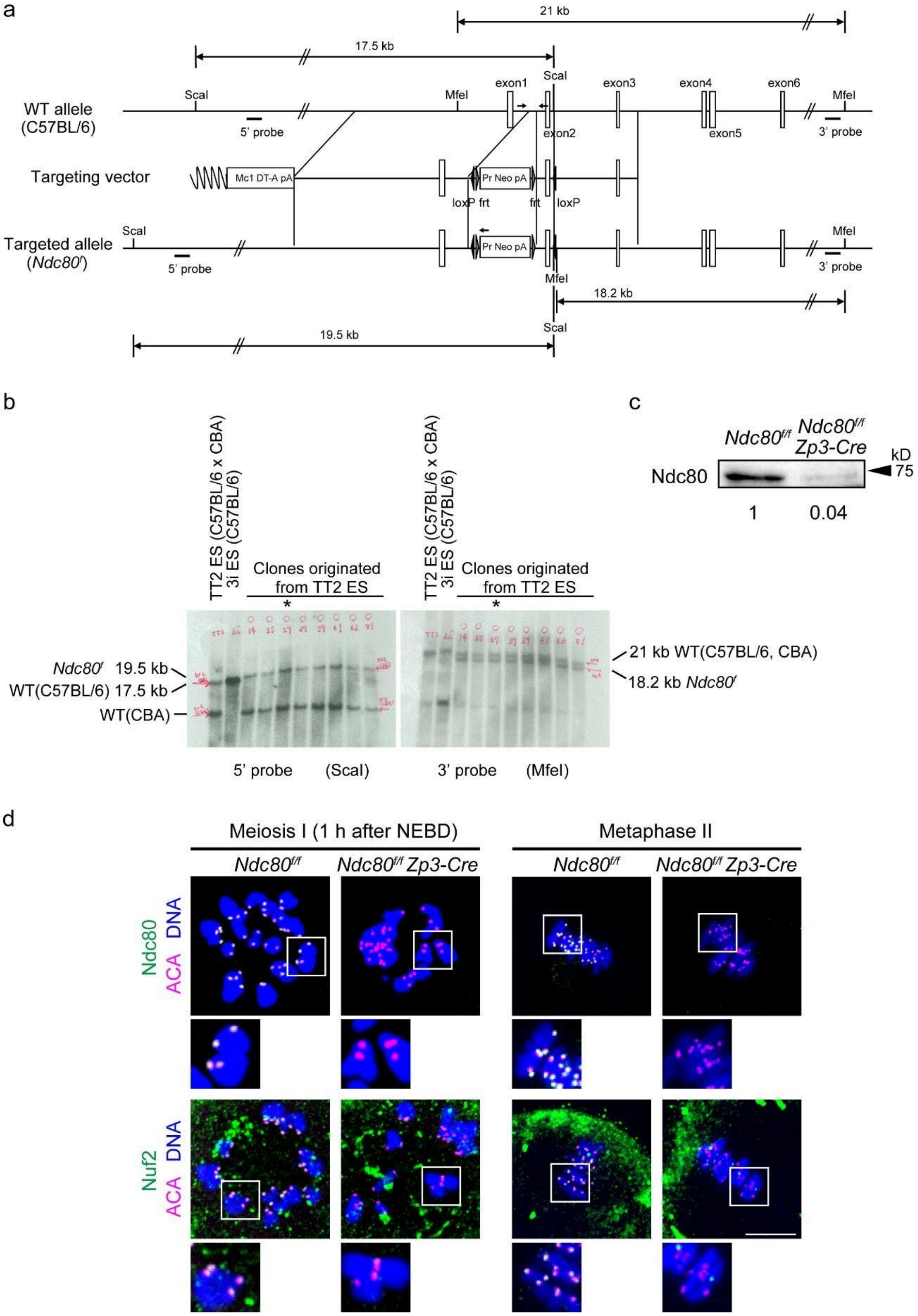

Supplementary Figure 1: Oocyte-specific deletion of the *Ndc80* gene.

a, *Ndc80* gene targeting. The scheme is drawn based on the genome sequence of the C57BL/6 allele. In the targeting vector, exon 2 of *Ndc80* was flanked by *loxP* sites with an *frt*-flanked neomycin (Neo) selection cassette. The positions of the probes and restriction enzymes used in the Southern blot analysis presented in (B) are shown. Arrows indicate the positions of the primers used for genotyping (Ndc80-fw, Ndc80-wt-rev, and Neo-rev; see Methods). **b**, Southern blot analysis. The targeting vector was introduced into TT2 ES cells (C57BL/6 × CBA). After neomycin selection, the genomes of the ES clones were digested by ScaI and MfeI, and tested with the 5' and 3' probes, respectively. Note that in the original TT2 ES cells, the 5' probe detected a 17.5 kb band and an additional more rapidly migrating band. The 17.5 kb band originated from the C57BL/6 allele, while the faster band was produced due to an extra ScaI site in the CBA allele (compare the lanes labeled 'TT2 ES (C57BL/6 × CBA)' and '3i ES (C57BL/6)'). All positive ES clones (No. 14, 25, 27, 37, 39, 61, 63, and 71) exhibited a 19.5 kb band with the 5' probe and an 18.2 kb band with the 3' probe, which is consistent with proper integration of the targeting DNA into the C57BL/6 allele. The *Ndc80$^{f/f}$* mice used in this study originated from clone 27, which is indicated by asterisks. The proper insertion was repeatedly confirmed by every PCR genotyping of the resultant mouse line.

c, Western blot analysis. Fully-grown oocytes at the GV stage were collected from *Ndc80$^{f/f}$* and *Ndc80$^{f/f}$ Zp3-Cre* mice, and their extracts were tested by Western blotting analysis with the Ndc80 antibody. The same number of oocytes (120 collected from 5 mice) was used for each sample. The relative intensity of the Ndc80 bands is shown. A full scan image is provided in the Source Data file.

d, Immunostaining. Oocytes at the prometaphase I stage (1 h after NEBD) and metaphase II stage (16 h after NEBD) were fixed and stained for Ndc80 or Nuf2 (green), kinetochores (ACA, magenta), and DNA (Hoechst33342, blue). Magnified images are shown. Ndc80 was undetectable at kinetochores in 20 MI oocytes and 21 MII oocytes. Nuf2, which requires Ndc80 for its kinetochore localization [28–30], was undetectable at kinetochores in 6 MI oocytes and 6 MII oocytes. Four independent experiments for Ndc80 and three independent experiments for Nuf2 were performed. Scale bar, 10 μm.

Supplementary Figure 2

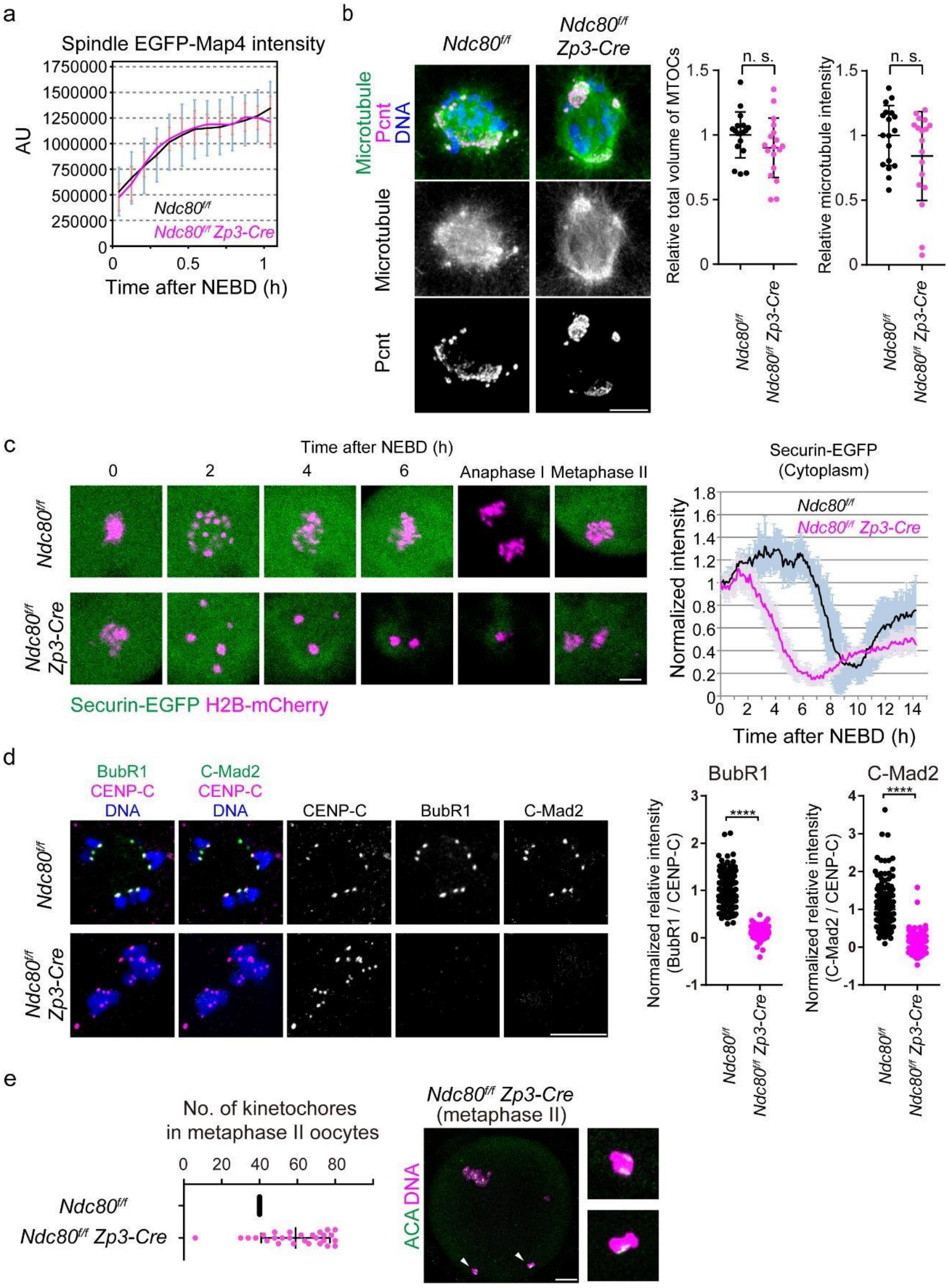

Supplementary Figure 2: *Ndc80*-deleted oocytes undergo intact microtubule nucleation and precocious anaphase I.

a, Initial microtubule nucleation does not require Ndc80. The kinetics of the increase in EGFPMap4 signals (microtubules) upon NEBD are shown (n = 9, 7 oocytes). **b,** Microtubule density and MTOC volume are largely intact in *Ndc80*-deleted oocytes. Oocytes 1 h after NEBD were immunostained for microtubules (anti-tubulin, green), MTOCs (antipericentrin, Pcnt, magenta) and counterstained for DNA (Hoechst33342, blue). Measurements were performed after 3D reconstruction (n = 18, 18 oocytes from 3 independent experiments). n.s., not significant by two-tailed unpaired Student's t-test.

c, Precocious entry to anaphase I in *Ndc80*-deleted oocytes. *Ndc80$^{f/f}$ Zp3-Cre* and *Ndc80$^{f/f}$* oocytes expressing Securin-EGFP (green) and H2B-mCherry (chromosomes, magenta) were monitored. Securin degradation is a marker for anaphase entry. Precocious securin degradation in *Ndc80*-deficient oocytes is consistent with spindle checkpoint defects [42,54,55]. Time after NEBD. Securin-EGFP levels in the cytoplasm were measured and plotted (n = 9, 9 oocytes).

d, Spindle checkpoint defects in *Ndc80*-deleted oocytes. Oocytes 1 h after NEBD were immunostained for BubR1 (green), active form Mad2 (C-Mad2, green), CENP-C (magenta), and counterstained for DNA (Hoechst33342, blue). In the plot, spots correspond to individual kinetochores (n = 200, 194 kinetochores from 5, 5 oocytes). ****$p<0.0001$ ($p=9.0E-120$ for BubR1, $p=3.06E-59$ for C-Mad2) by two-tailed unpaired Student's t-test.

e, Aneuploidy in *Ndc80*-deleted oocytes. Oocytes at metaphase II were immunostained for kinetochores (ACA, green) and counterstained for DNA (Hoechst33342, magenta). The number of kinetochores were counted (n = 28, 28 oocytes from 3 independents experiments). The square indicates a chromosome carrying four kinetochores (arrowheads in the magnified image), presumably derived from chromosome nondisjunction at anaphase I. For an entire set of z-slice images, please see Supplementary Fig. 3 and Supplementary Movie 4.

Scale bars, 10 µm. Mean +/- SD are presented in (a) (b) (c) (d) and (e).

Supplementary Figure 3

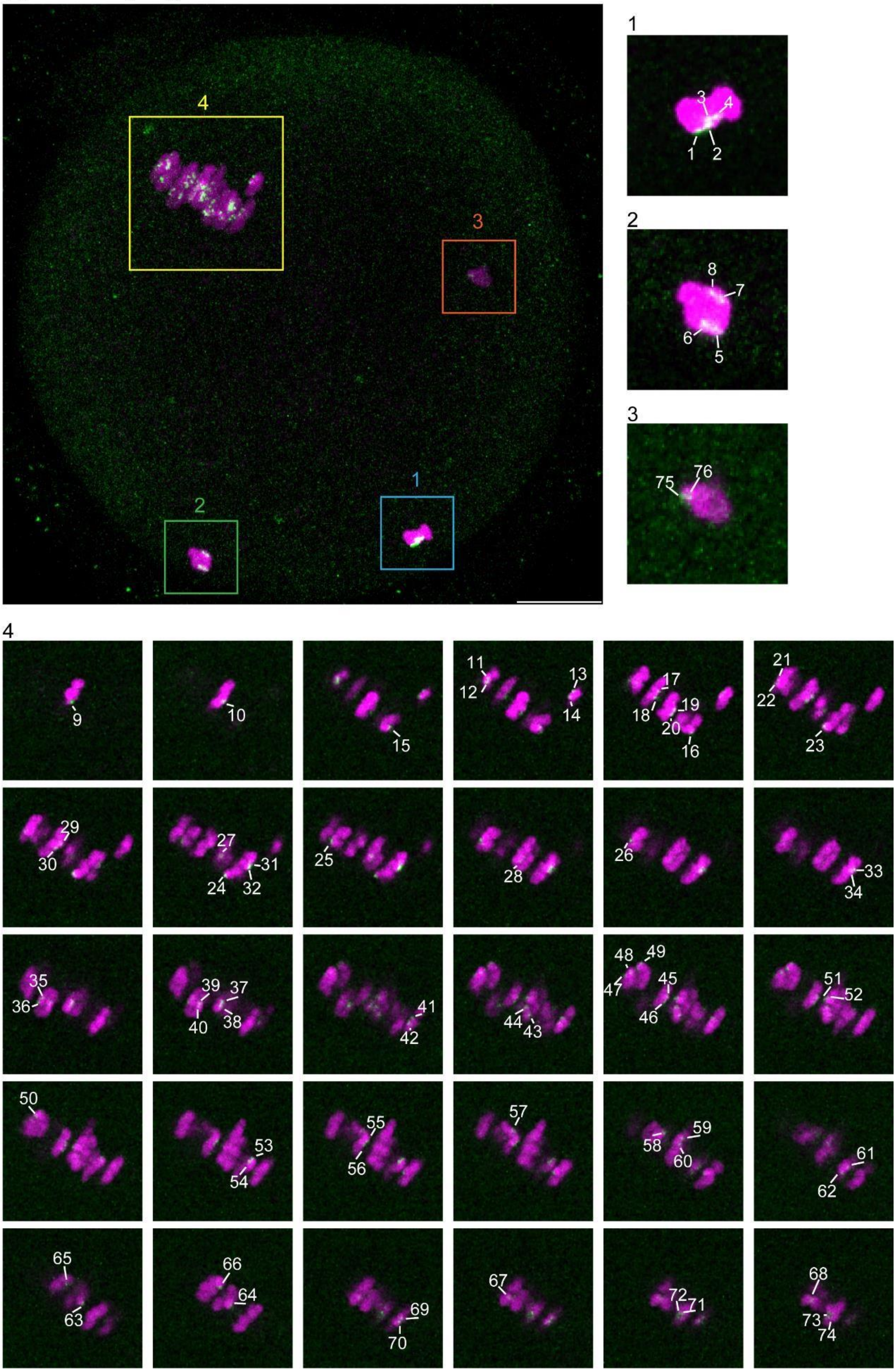

Supplementary Figure 3: Kinetochore counting in MII eggs.

Oocytes at metaphase II were immunostained for kinetochores (ACA, green) and counterstained for DNA (Hoechst33342, magenta). Optical slice images in a z-stack of *Ndc80$^{f/f}$ Zp3-Cre* oocytes are shown. Numbers indicate kinetochore counts. Three independent experiments were performed. Scale bar, 10 µm. See also Supplementary Movie 4.

Supplementary Figure 4

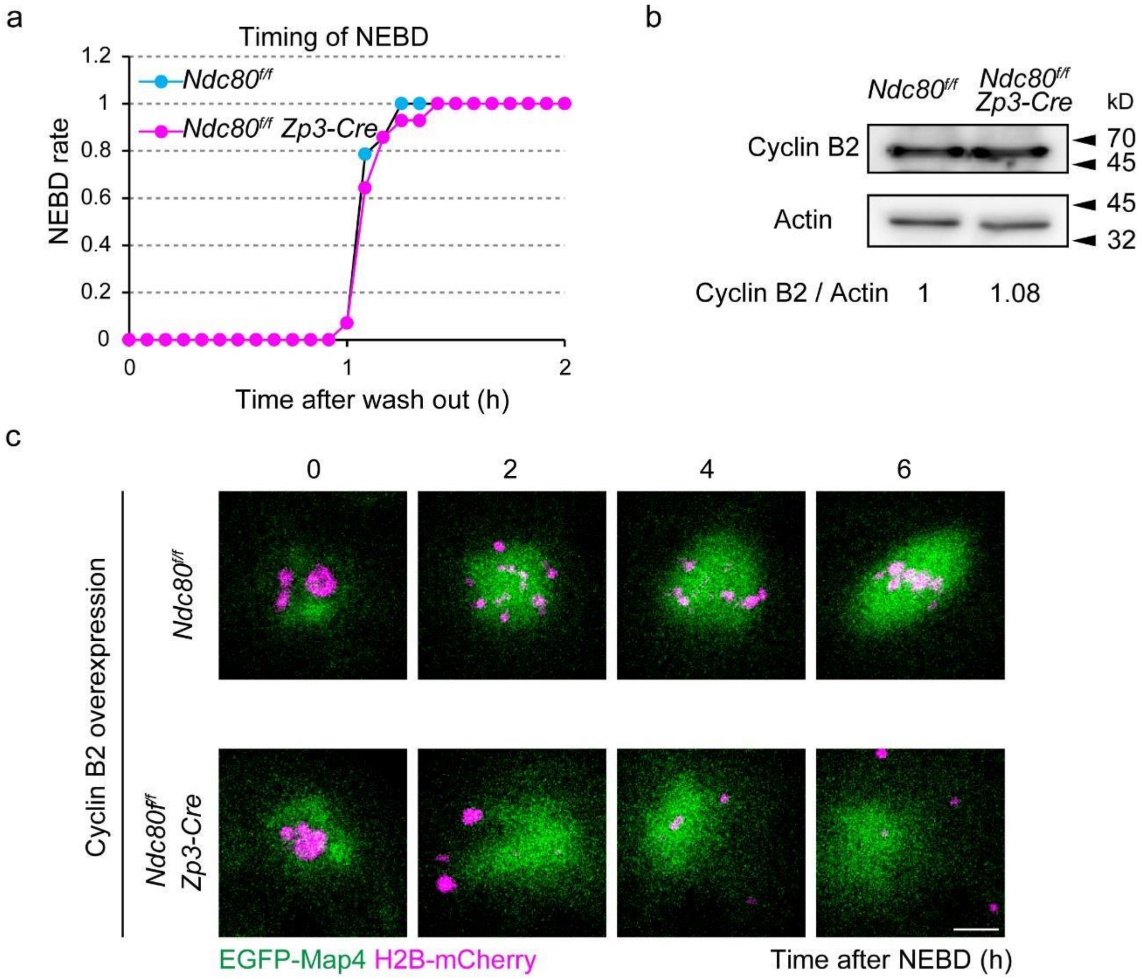

Supplementary Figure 4: Cyclin B2 is intact in *Ndc80*-deleted oocytes.

a, No delay in NEBD in *Ndc80*-deleted oocytes. *Ndc80$^{f/f}$ Zp3-Cre* and *Ndc80$^{f/f}$* oocytes were monitored after the induction of meiotic resumption. The fraction of oocytes that underwent NEBD was plotted (n = 14, 14). Three independent experiments were performed.

b, No decrease in cyclin B2 levels. Extracts from *Ndc80$^{f/f}$ Zp3-Cre* and *Ndc80$^{f/f}$* oocytes at the GV stage were tested for cyclin B2 levels. The same number of oocytes (50 oocytes) was used for each sample. After stripping of cyclin B2 antibody, actin was detected using the same membrane. The signal intensities of cyclin B2 relative to actin are shown. Full scan images are provided in the Source Data file.

c, Cyclin B2 overexpression does not rescue spindle defects. Cyclin B2 RNAs were comicroinjected with RNAs encoding EGFP-Map4 (microtubules, green) and H2B-mCherry (chromosomes, magenta) into *Ndc80$^{f/f}$ Zp3-Cre* and *Ndc80$^{f/f}$* oocytes at the GV stage. These

oocytes underwent NEBD despite the presence of the meiotic resumption inhibitor IBMX, indicating effective cyclin B2 overexpression. Nevertheless, the spindle defects in *Ndc80$^{f/f}$ Zp3-Cre* oocytes were not rescued. Five oocytes in each group were observed. Scale bar, 10 μm.

Supplementary Figure 5

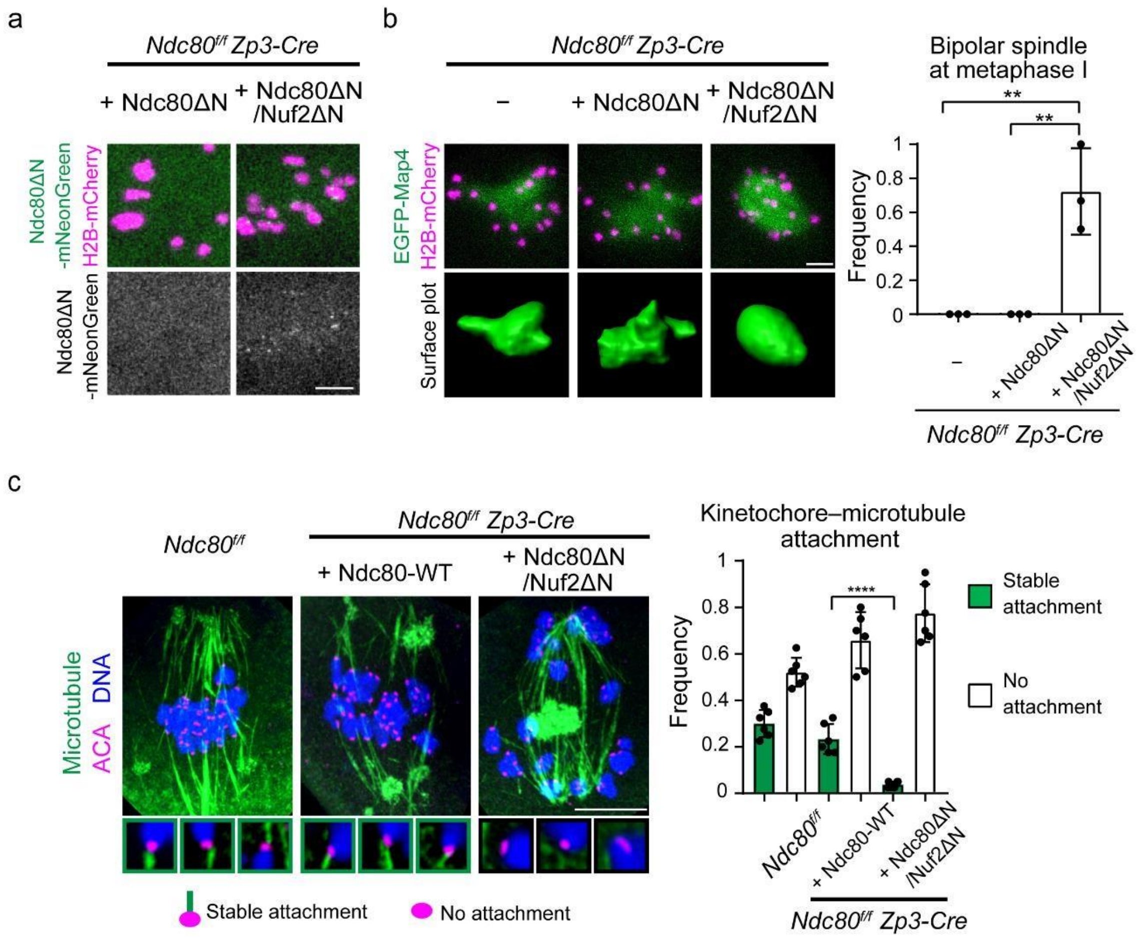

Supplementary Figure 5: Ndc80ΔN requires Nuf2ΔN to localize to kinetochores and to rescue spindle bipolarization.

a, Ndc80ΔN requires Nuf2ΔN for kinetochore localization. *Ndc80$^{f/f}$ Zp3-Cre* oocytes expressing Ndc80ΔN-mNeonGreen (green) and H2B-mCherry (chromosomes, magenta) with or without Nuf2ΔN were monitored (n = 6, 6 oocytes). Note that the kinetochore localization of Ndc80ΔN was detected only when coexpressed with Nuf2ΔN.

b, Ndc80ΔN requires Nuf2ΔN for spindle rescue. *Ndc80$^{f/f}$ Zp3-Cre* oocytes expressing EGFPMap4 (microtubules, green), H2B-mCherry (chromosomes, magenta), and Ndc80ΔN with or without Nuf2ΔN were monitored. Note that spindle bipolarization was rescued only when Ndc80ΔN and Nuf2ΔN were coexpressed. Spindle shapes reconstructed into 3D at metaphase I (5.5 h after NEBD) were categorized based on the aspect ratio and surface irregularity. The frequency of oocytes with a bipolar-shaped spindle is shown (n = 15, 16, 15 oocytes from 3 independent experiments). **p = 0.0080, **p = 0.0080 by two-tailed unpaired Student's t-test. **c**, Ndc80ΔN/Nuf2ΔN-rescued spindles fail to establish kinetochore–microtubule attachment. Oocytes at metaphase I (5.5 h after NEBD) were briefly treated with a cold buffer prior to fixation

for visualization of kinetochore–microtubule attachments. Microtubules (green), kinetochores (ACA, magenta), and DNA (Hoechst33342, blue) are shown. All 40 kinetochores in each oocyte were analyzed (n = 6, 6, 6 oocytes from 3 independent experiments). Kinetochores exhibiting amphitelic microtubule attachment were categorized as ‘Stable attachment’. Kinetochores with no associated microtubules were categorized as ‘No attachment’. ****$p<0.0001$ ($p=2.7E-05$) by two-tailed unpaired Student’s t-test.

Scale bars, 10 μm. Mean +/- SD are presented in (b) and (c).

Supplementary Figure 6

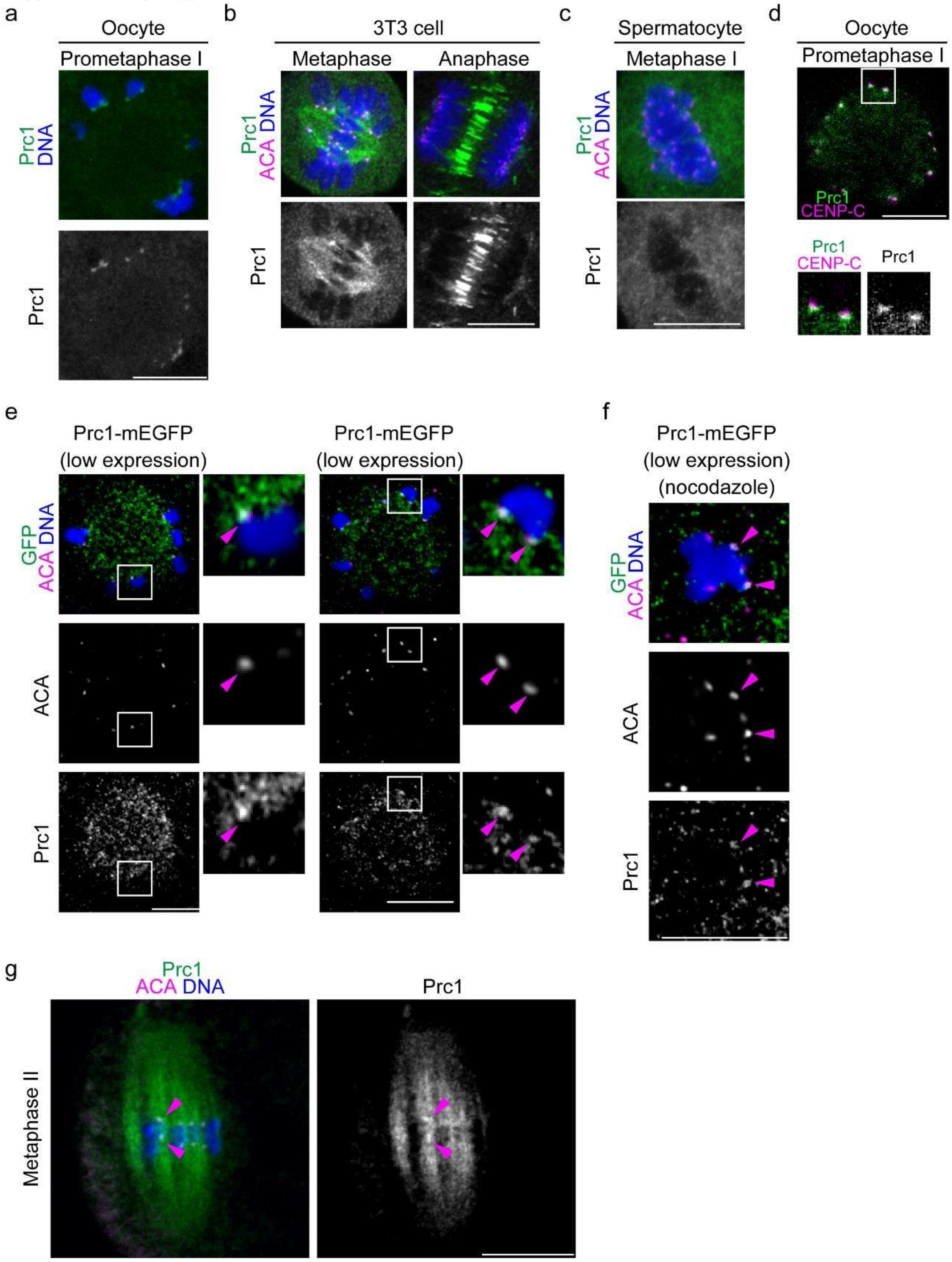

Supplementary Figure 6: Prc1 is detected at kinetochores in oocytes but not in cultured centrosomal cells or in spermatocytes.

a, Prc1 localizes at kinetochores in oocytes. Oocytes 2 h after NEBD were immunostained for Prc1 (anti-Prc1, green) and counterstained for DNA (Hoechst33342, blue). Three independent experiments were performed.

b, Prc1 localizations in centrosomal cells during mitosis. NIH3T3 cells were fixed and immunostained for Prc1 (green), kinetochores (ACA, magenta) and DNA (Hoechst33342, blue). Prc1 was detected around spindle poles during prometaphase and at the spindle midzone during anaphase, but not at kinetochores. Three independent experiments were performed.

c, Prc1 is not enriched at kinetochores in spermatocytes during meiosis I. Spermatocytes at metaphase I were fixed and immunostained for Prc1 (green), kinetochores (ACA, magenta) and DNA (Hoechst33342, blue). Three independent experiments were performed.

d, SunTag-Prc1 is enriched at kinetochores in oocytes. SunTag-Prc1 (24xGCN4-Prc1 coexpressed with scFv-sfGFP, green) signals at kinetochores (labeled with tdTomato-CENP-C, magenta) in live oocytes were shown. More than three independent experiments were performed.

e and f, Prc1-mEGFP localizes at kinetochores in oocytes. Control or nocodazole-treated oocytes expressing Prc1-mEGFP were fixed at 2 h after NEBD and immunostained with anti-GFP (green) and DNA (Hoechst33342, blue). Note that we expressed Prc1-mEGFP at low levels to avoid overexpression artifacts of GFP-fused Prc1[1]. Three independent experiments were performed.

g, Prc1 localizes at kinetochores and microtubules at metaphase II. One of the z-slice images of Fig. 2b at metaphase II is shown. Arrowheads indicate kinetochores. More than three independent experiments were performed.

Scale bars, 10 μm.

Supplementary Figure 7

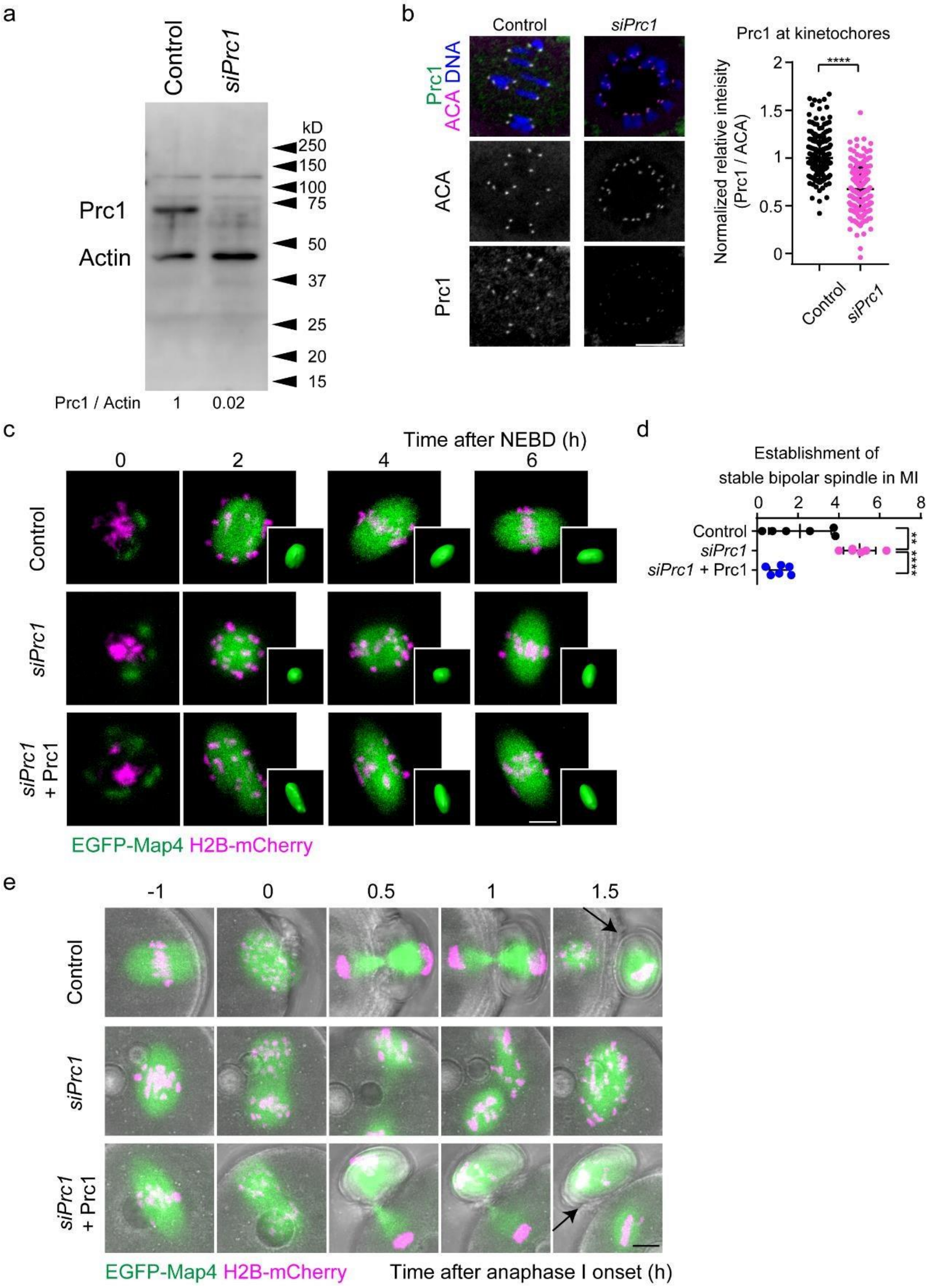

Supplementary Figure 7: Validation of Prc1 RNAi.

a, Depletion of Prc1 by RNAi. Oocytes microinjected with an siRNA targeting *Prc1* (*siPrc1*) or an siRNA targeting luciferase (control) were collected at metaphase I (6 h after NEBD) for Western blotting. Fifty oocytes were used for each sample. Three independent experiments were performed. A full scan image is provided in the Source Data file.

b, Reduced kinetochore Prc1 by RNAi. Oocytes microinjected with an siRNA targeting Prc1 (*siPrc1*) or luciferase (control) were fixed 2 h after NEBD. Oocytes were immunostained for Prc1 (green), ACA (kinetochores, magenta), and DNA (Hoechst33342, blue). In the plot, spots correspond to individual kinetochores and oocytes, respectively (n = 200, 200 kinetochores from 5, 5 oocytes). ****p<0.0001 (p=1.7E-38) by two-tailed unpaired Student's t-test. **c**, Live imaging of oocytes after Prc1 RNAi. Oocytes were labeled with EGFP-Map4 (microtubules, green) and H2B-mCherry (chromosomes, magenta). To rescue Prc1 RNAi, siRNA-resistant Prc1 was expressed (+ Prc1). Spindle shapes were reconstructed in 3D. Three independent experiments were performed.

d, Prc1 depletion delays spindle bipolarization in MI. Spindle shapes in 3D were categorized based on the aspect ratio, surface irregularity, and stability (see Methods). The plot shows the time at which a stable bipolar spindle was established (n = 6, 6, 6 oocytes). **p = 0.002, ****p<0.0001 (p=1.2E-06) by two-tailed unpaired Student's t-test.

e, Prc1 RNAi causes defects in cytokinesis. Images of oocytes expressing EGFP-Map4 (microtubules, green) and H2B-mCherry (chromosomes, magenta) after Prc1 RNAi with or without expression of siRNA-resistant Prc1 are shown. Arrows indicate polar bodies. Oocytes following Prc1 RNAi failed polar body extrusion, consistent with the role of Prc1 in cytokinesis reported in somatic cultured cells [56–58]. Three independent experiments were performed.

Scale bars, 10 μm. Mean +/- SD are presented in (b) and (d).

Supplementary Figure 8

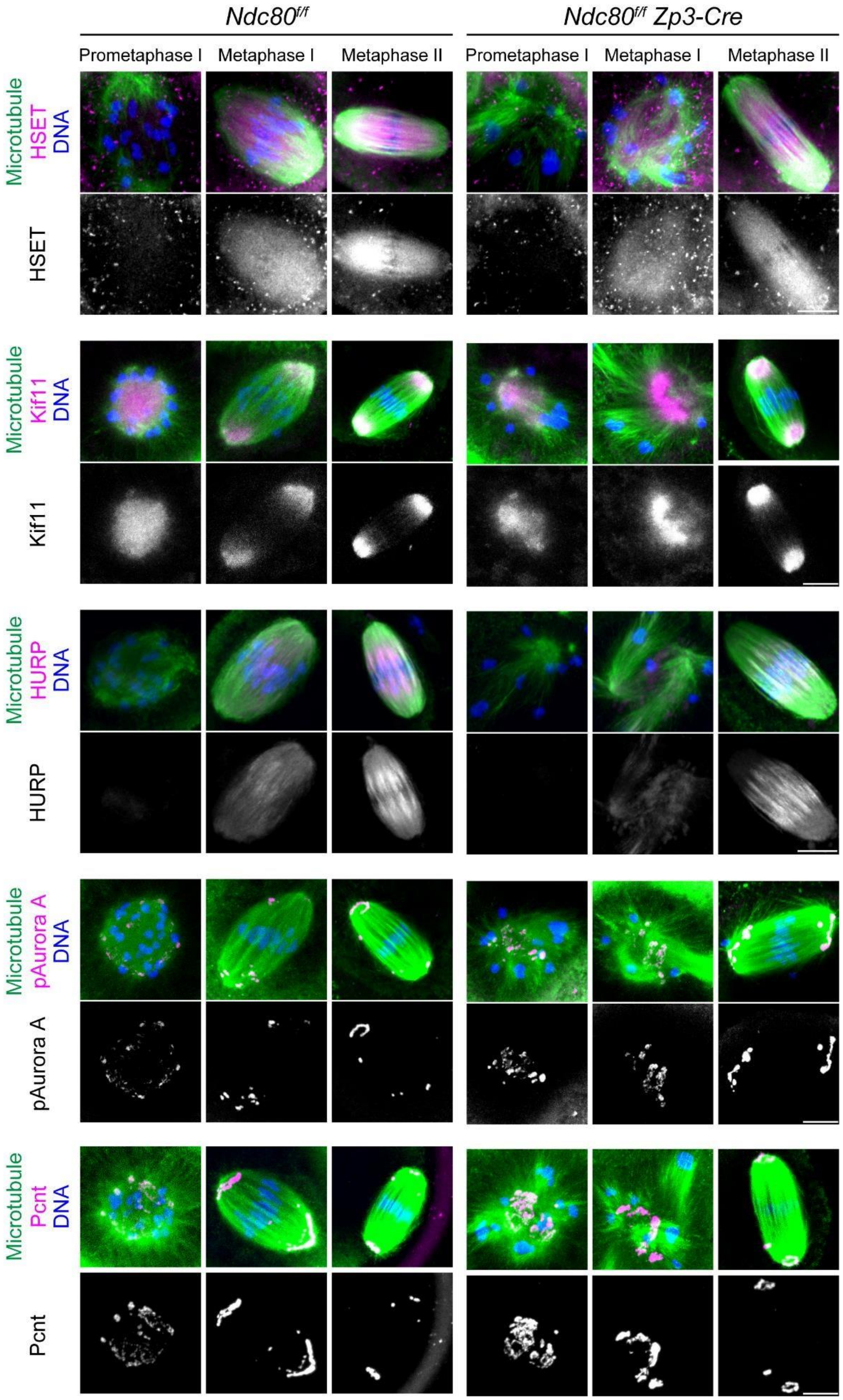

Supplementary Figure 8: *Ndc80* deletion does not directly affect the localization of HSET, Kif11, HURP, pAurora A or pericentrin.

Oocytes 2 h (Prometaphase I), 5 h (Metaphase I), 16 h (Metaphase II) after NEBD were immunostained for HSET, Kif11, HURP, phosphorylated Aurora A (pAurora A), or pericentrin (Pcnt), tubulin (microtubules, green), and DNA (Hoechst33342, blue). Three independent experiments were performed. Scale bars, 10 μm.

Supplementary Figure 9

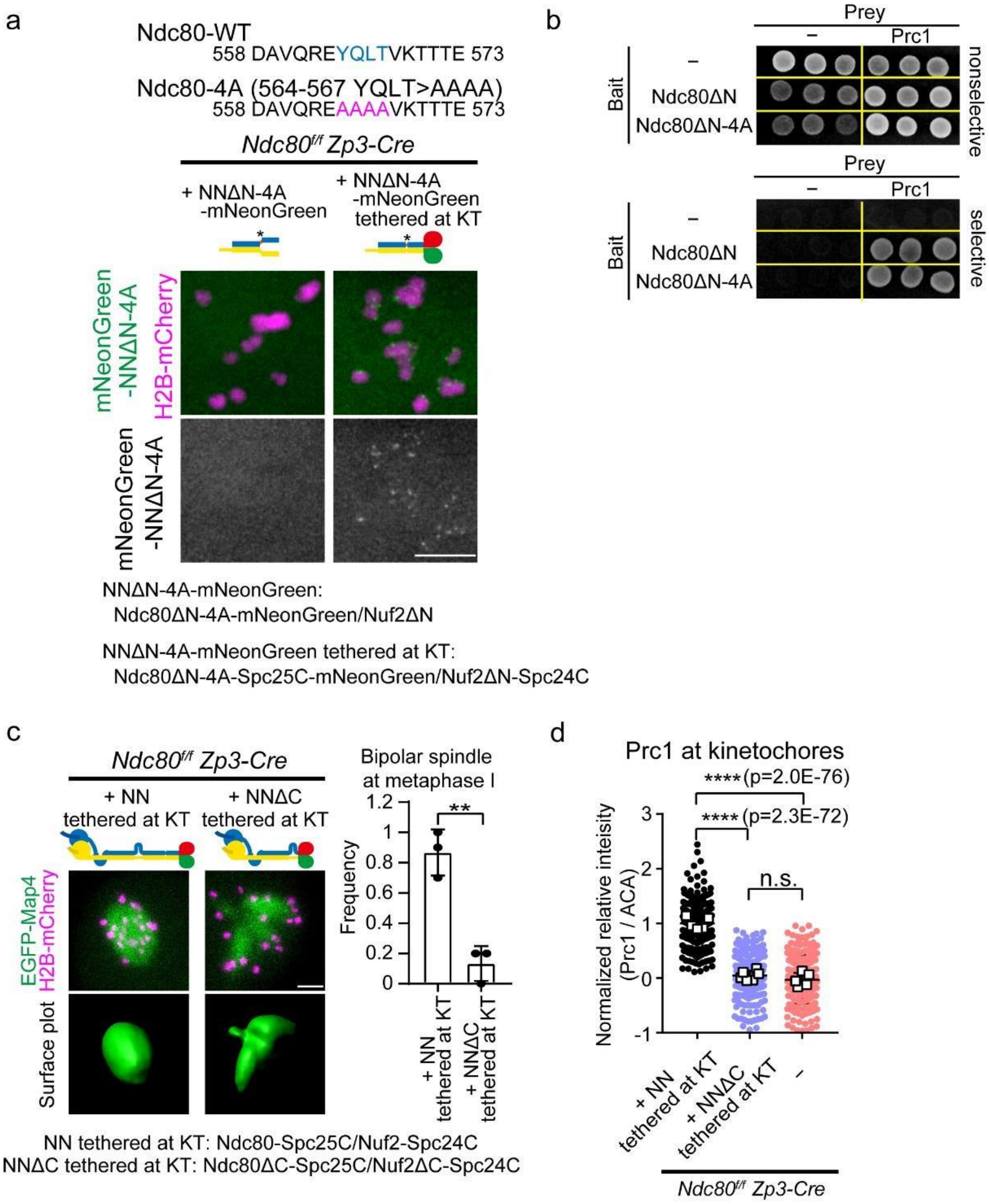

Supplementary Figure 9: Ndc80ΔN-4A/Nuf2ΔN acts at kinetochores for spindle bipolarization.

a, *Ndc80$^{f/f}$ Zp3-Cre* oocytes expressing Ndc80ΔN-4A-mNeonGreen/Nuf2ΔN (indicated as 'NNΔN-4A-mNeonGreen'), or Ndc80ΔN-4A-Spc25C-mNeonGreen/Nuf2ΔN-Spc24C (indicated as 'NNΔN-4A-mNeonGreen tethered at KT') with H2B-mCherry (chromosomes, magenta) were monitored (n = 3, 3 oocytes). Images from 3 h after NEBD are shown. Note that Ndc80ΔN-4AmNeonGreen/Nuf2ΔN failed to localize to kinetochores. Kinetochore localization was

recovered when the construct was fused to the kinetochore-targeting domains Spc25C and Spc24C.

b, Ndc80ΔN-4A retains the capacity to interact with Prc1. Yeast two-hybrid assay using selective (−His, −Ade) and nonselective (+His, +Ade) plates.

c, Ndc80/Nuf2 requires the Prc1 binding domain for spindle bipolarization. *Ndc80^f/f^ Zp3-Cre* oocytes expressing Ndc80-Spc25C/Nuf2-Spc24C (indicated as 'NN tethered at KT') or Ndc80ΔC-Spc25C/Nuf2ΔC-Spc24C (indicated as 'NNΔC tethered at KT') were monitored. Ndc80ΔC (a.a. 1–453) and Nuf2ΔC (a.a. 1–287) lack Prc1-binding domains. Spindle shapes were reconstructed in 3D and categorized based on the aspect ratio and surface irregularity. The frequency of oocytes that exhibited a bipolar-shaped spindle is shown (n = 26, 26 oocytes from 3 independent experiments). **p=0.0027 by two-tailed unpaired Student's t-test.

d, Prc1-interacting domains of Ndc80/Nuf2 are required for Prc1 recruitment at kinetochores. *Ndc80^f/f^ Zp3-Cre* oocytes expressing Ndc80-Spc25C/Nuf2-Spc24C (indicated as 'NN tethered at KT') or Ndc80ΔC-Spc25C/Nuf2ΔC-Spc24C (indicated as 'NNΔC tethered at KT') were immunostained for Prc1, kinetochores, and DNA. Ndc80ΔC (a.a. 1–453) and Nuf2ΔC (a.a. 1–287) lack Prc1-binding domains. In the plot, spots and squares correspond to individual kinetochores and oocytes, respectively (n = 200, 194, 200 kinetochores from 5, 6, 5 oocytes). ****p<0.0001 (exact values are shown in the panel) by two-tailed unpaired Student's t-test. n.s., not significant.

Scale bars, 10 µm. Mean +/- SD are presented in (c) and (d).

Supplementary Figure 10

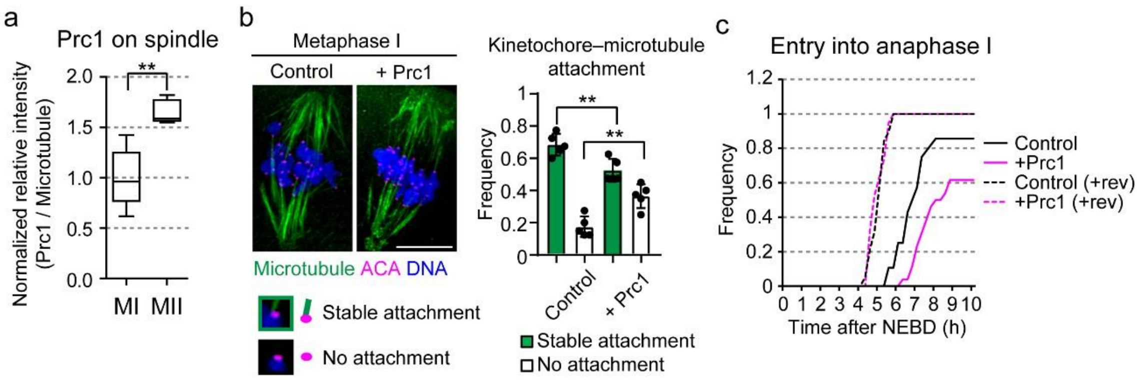

Supplementary Figure 10: An artificial increase in Prc1 expression delays anaphase I onset in a spindle checkpoint-dependent manner.

a, Prc1 is increased in MII spindles. Prc1 signals along spindle microtubules at metaphase I (6 h after NEBD) and metaphase II (16 h after NEBD) were measured (n = 5, 5 oocytes). Boxes show the 25th to 75th percentiles and whiskers show the minimum to the maximum. **p<0.0016 by twotailed unpaired Student's t-test. For images, see Fig. 2b.

b, Increased Prc1 decreases kinetochore–microtubule attachment. Oocytes at 6 h after NEBD were briefly treated with a cold buffer prior to fixation for visualization of kinetochore– microtubule attachments. Microtubules (green), kinetochores (ACA, magenta), and DNA (Hoechst33342, blue) are shown. All 40 kinetochores in each oocyte were analyzed (n = 6, 6, 6 oocytes from 3 independent experiments). Kinetochores exhibiting amphitelic microtubule attachment were categorized as 'Stable attachment'. Kinetochores with no associated microtubules were categorized as 'No attachment'. **p = 0.0064 (Stable attachment), **p = 0.0025 (No attachment) by two-tailed unpaired Student's t-test. Scale bar, 10 μm. Mean +/- SD are presented in (b) and (d). **c**, An artificial increase in Prc1 causes a spindle checkpoint-dependent delay in anaphase I onset. Oocytes expressing increased levels of Prc1 were cultured in the presence of the spindle checkpoint inhibitor reversine (rev) and monitored for anaphase I onset. The percentages of oocytes that underwent anaphase I onset were plotted (n = 28, 26, 18, 19 oocytes from 3 independent experiments).

Supplementary Figure 11

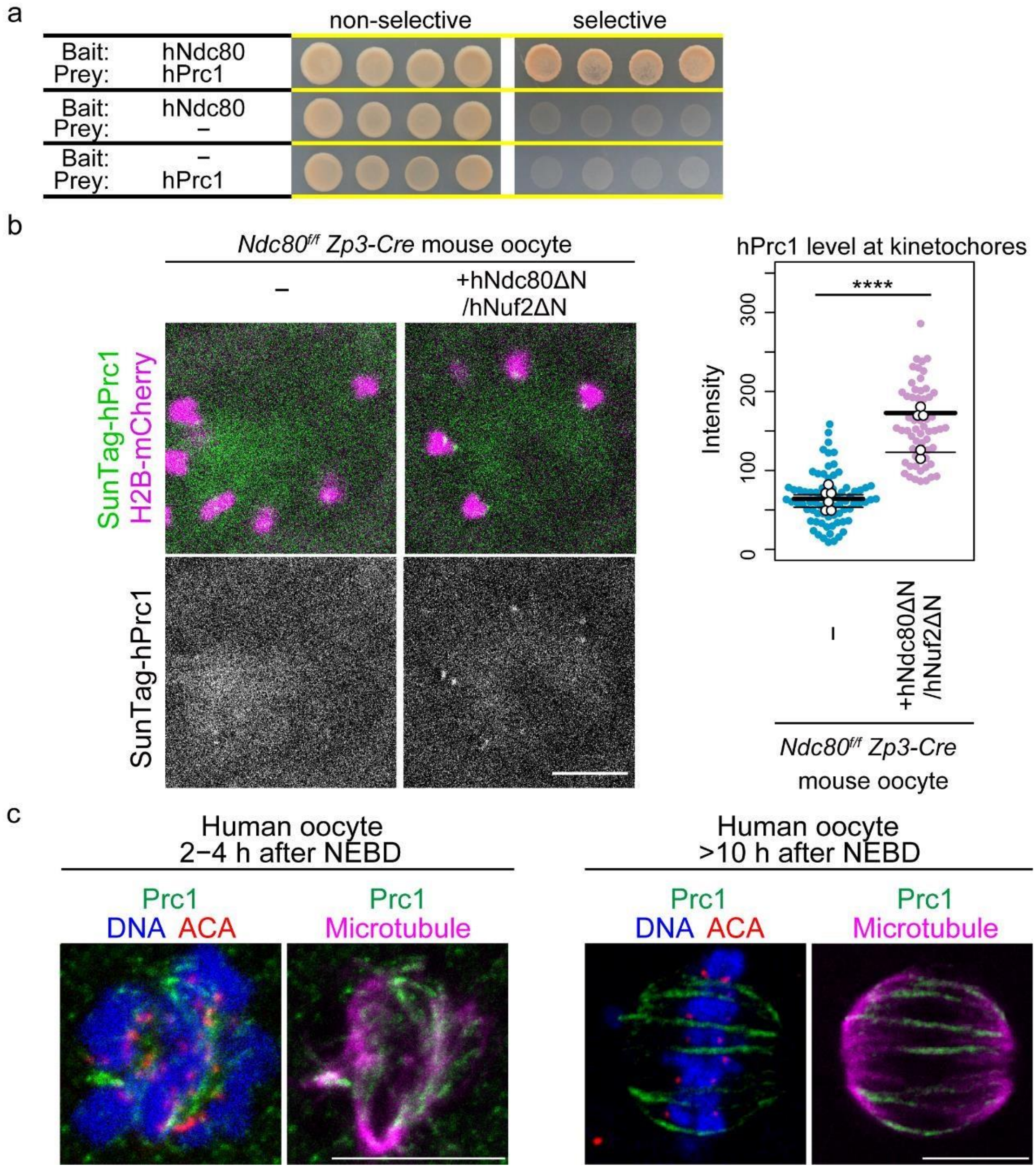

Supplementary Figure 11: Human oocytes do not exhibit detectable Prc1 enrichment at kinetochores.

a, Human Ndc80 can interact with human Prc1. Yeast two-hybrid assay using selective (−His, −Ade) and nonselective (+His, +Ade) plates. A fragment of human Prc1 (a.a. 96–304) and fulllength human Ndc80 were tested.

b, Human Ndc80 recruits human Prc1 to kinetochores in mouse oocytes. SunTag-human Prc1 (24xGCN4-human Prc1 coexpressed with scFv-sfGFP [67], green) and H2B−mCherry (chromosomes, magenta) were monitored in *Ndc80$^{f/f}$ Zp3-Cre* mouse oocytes expressing human

Ndc80ΔN (aa. 461–641) and Nuf2ΔN (a.a. 276–464). In the plot, spots and squares correspond to individual kinetochores and oocytes, respectively (n = 60, 50 kinetochores from 6, 5 oocytes). Bars indicate 25%, 50% (median), and 75% percentiles. ****$p<0.0001$ ($p=2.2E-19$) by two-tailed unpaired Student's t-test.

c, Prc1 localizes along spindle microtubules but not at kinetochores in human oocytes. Human oocytes, which were retarded in maturation, were fixed at indicated timings and immunostained for Prc1 (green), ACA (kinetochores, red), microtubules (magenta) and DNA (Hoechst33342, blue). Oocytes from more than three women were examined.

Scale bars, 10 μm.

Supplementary Figure 12

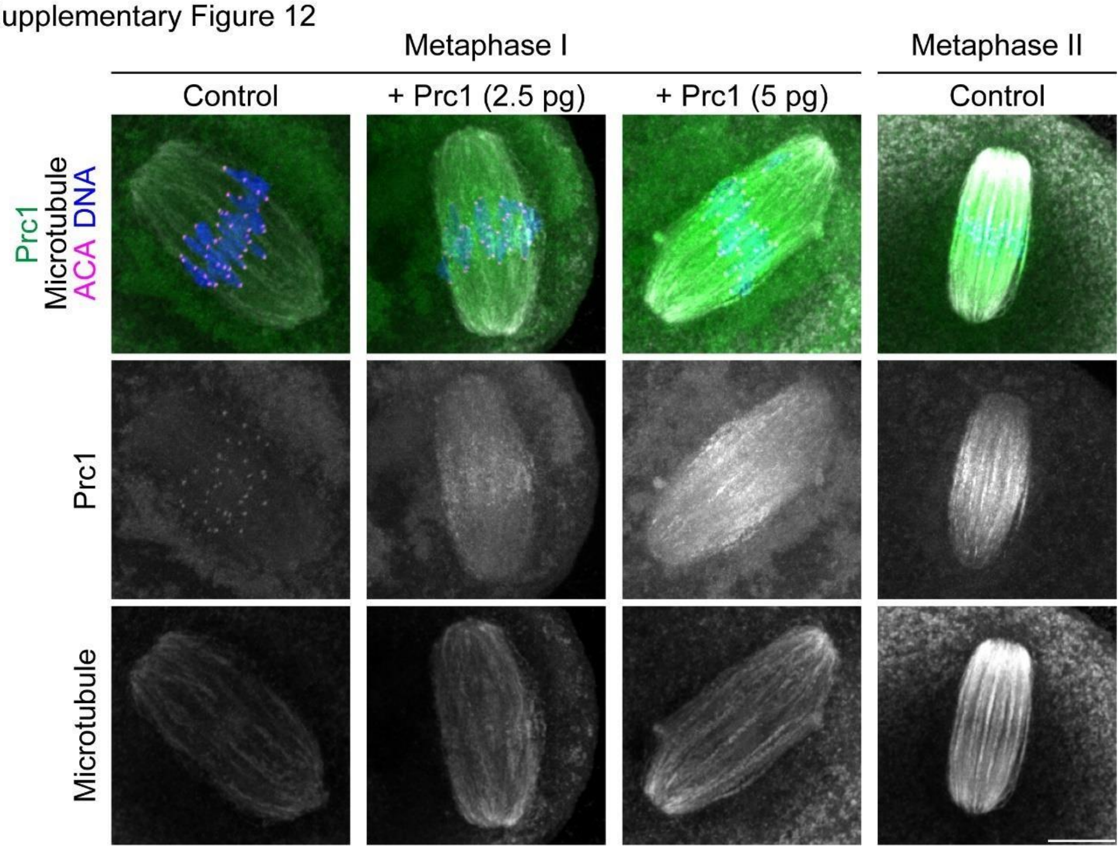

Supplementary Figure 12: Increased Prc1 expression promotes its localization to spindle microtubules.

Oocytes were microinjected with 2.5 or 5 pg mRNAs of Prc1. The oocytes were immunostained for Prc1 (green), ACA (kinetochores, magenta), tubulin (microtubules, gray), and DNA (Hoechst33342, blue) at metaphase I and metaphase II. Increase of spindle Prc1 by Prc1 overexpression was examined by more than three independent experiments. Scale bar, 10 μm.

Supplementary Reference

1. Hu, C.-K., Özlü, N., Coughlin, M., Steen, J. & Mitchison, T. Plk1 negatively regulates PRC1 to prevent premature midzone formation before cytokinesis. *Mol. Biol. Cell* **23**, 2702–2711 (2012).
