# Supplementary material for: Prc1-rich kinetochores are required for error-free acentrosomal spindle bipolarization during meiosis I in mouse oocytes
Source: Nat Commun. 2020 May 27;11:2652. doi: 10.1038/s41467-020-16488-y (PMC7253481; doi:10.1038/s41467-020-16488-y)
Supplement: Supplementary file 13 — Reporting Summary [file 41467_2020_16488_MOESM13_ESM.pdf]

nature research

Corresponding author(s): Tomoya S. Kitajima

Last updated by author(s): Apr 21, 2020

Reporting Summary

Nature Research wishes to improve the reproducibility of the work that we publish. This form provides structure for consistency and transparency in reporting. For further information on Nature Research policies, see Authors & Referees and the Editorial Policy Checklist.

Statistics

For all statistical analyses, confirm that the following items are present in the figure legend, table legend, main text, or Methods section.

n/a	Confirmed	
☐	☒	The exact sample size (*n*) for each experimental group/condition, given as a discrete number and unit of measurement
☐	☒	A statement on whether measurements were taken from distinct samples or whether the same sample was measured repeatedly
☐	☒	The statistical test(s) used AND whether they are one- or two-sided<br>*Only common tests should be described solely by name; describe more complex techniques in the Methods section.*
☒	☐	A description of all covariates tested
☒	☐	A description of any assumptions or corrections, such as tests of normality and adjustment for multiple comparisons
☐	☒	A full description of the statistical parameters including central tendency (e.g. means) or other basic estimates (e.g. regression coefficient) AND variation (e.g. standard deviation) or associated estimates of uncertainty (e.g. confidence intervals)
☐	☒	For null hypothesis testing, the test statistic (e.g. F, t, r) with confidence intervals, effect sizes, degrees of freedom and P value noted<br>*Give P values as exact values whenever suitable.*
☒	☐	For Bayesian analysis, information on the choice of priors and Markov chain Monte Carlo settings
☒	☐	For hierarchical and complex designs, identification of the appropriate level for tests and full reporting of outcomes
☒	☐	Estimates of effect sizes (e.g. Cohen's *d*, Pearson's *r*), indicating how they were calculated

Our web collection on statistics for biologists contains articles on many of the points above.

Software and code

Policy information about availability of computer code

Data collection	Zen Software (Carl Zeiss) version ZEN 2011 SP3 (black) version 8.1.11.484 for Lsm710 and Lsm780, ZEN 2.3 SP1 FP1 (black) version 14.0.13.201 and ZEN2.3 SP1 FP1 (black) version 14.0.9.201 for Lsm880 were used to capture confocal images. Fiji (https://fiji.sc/) was used to quantify fluorescent signals. To analyze spindle shapes, we performed 3D surface rendering of the signals of EGFP-Map4 or microtubules with Imaris software (Bitplane) version 7.4.2.
Data analysis	Statistical analyses were performed using Excel version 2016 and GraphPad Prism version 7.02.

For manuscripts utilizing custom algorithms or software that are central to the research but not yet described in published literature, software must be made available to editors/reviewers. We strongly encourage code deposition in a community repository (e.g. GitHub). See the Nature Research guidelines for submitting code & software for further information.

Data

Policy information about availability of data

All manuscripts must include a data availability statement. This statement should provide the following information, where applicable:

- Accession codes, unique identifiers, or web links for publicly available datasets
- A list of figures that have associated raw data
- A description of any restrictions on data availability

All data of this study are stored at the corresponding author and available on reasonable request.

Field-specific reporting

Please select the one below that is the best fit for your research. If you are not sure, read the appropriate sections before making your selection.

☒ Life sciences ☐ Behavioural & social sciences ☐ Ecological, evolutionary & environmental sciences

For a reference copy of the document with all sections, see nature.com/documents/nr-reporting-summary-flat.pdf

Life sciences study design

All studies must disclose on these points even when the disclosure is negative.

Sample size	Sample size (number of oocytes) was chosen based on the number of fully grown oocytes obtained from one or two mice per experiment. The number of mice used in each experiment was limited to one or two. This limitation was set in order to carry out the procedures of oocyte collection within 30 minutes, which was required for ensuring the reproducibility of the results.
Data exclusions	No data exclusion in this study.
Replication	Experiments were repeated at least three times. All attempts at replication were successful.
Randomization	All samples were randomly allocated into different experimental groups.
Blinding	Investigators were not blinded. Blinding was technically difficult because experiments and analyses were done by same investigators.

Reporting for specific materials, systems and methods

We require information from authors about some types of materials, experimental systems and methods used in many studies. Here, indicate whether each material, system or method listed is relevant to your study. If you are not sure if a list item applies to your research, read the appropriate section before selecting a response.

Materials & experimental systems

n/a	Involved in the study	
☐	☒	Antibodies
☒	☐	Eukaryotic cell lines
☒	☐	Palaeontology
☐	☒	Animals and other organisms
☐	☒	Human research participants
☒	☐	Clinical data

Methods

n/a	Involved in the study	
☒	☐	ChIP-seq
☒	☐	Flow cytometry
☒	☐	MRI-based neuroimaging

Antibodies

Antibodies used	As primary antibodies:a rabbit anti-Ndc80 antiserum (1:500 for WB, 1:2000 for IF, a gift from Dr. Robert Benezra); a human anti-centromere antibodies (1:200 for oocyte, 1:500 for spermatocyte, ACA, , Europa Bioproducts); a rabbit anti-Nuf2 antibody (1:500, ab230313, Abcam); a mouse anti-α-tubulin antibody (1:500 for oocyte, 1:100 for culture cell, DM1A T6199, Sigma); a rat anti-GFP antibody (1:500, GF090R 04404-84, Nacalai); a rabbit anti-Prc1 (1:100 for oocyte, 1:500 for spermatocyte, H-70 sc-8356, Santa Cruz); a mouse anti-Ndc80 (1:500, 9G3.23, GeneTex); a rabbit anti-Aurora A (1:500, pT288) (NB100-2371, NOVUS biologicals); a sheep anti-BubR1 (1:100, ab28192, Abcam); a mouse anti-pericentrin (1:500, 611814, BD Transduction Laboratories); a rabbit anti-HURP (1:50, sc-98809, Santa Cruz); a mouse anti-Mad2 (1:500, sc-65492, Santa Cruz); a rabbit anti-HSET (1:200, a gift from Dr. Renata Basto); a mouse anti-GFP (1:500, ab1218, abcam); a rat anti-α-tubulin (1:2000, MCA77G, Bio-Rad); a rabbit anti-Kif11 (1:500, HPA010568, Sigma); a rabbit anti-CENP-C (1:500, a gift from Dr. Yoshinori Watanabe); a mouse anti-Scp3 (1:500, ab97672, Abcam); a goat anti-cyclin B2 (1:2000, AF6004, R&D Systems); and a rabbit anti-actin (1:2000, ab1801, Abcam).<br>As secondary antibodies: Alexa Fluor 488 goat anti-mouse IgG (H+L) (A11029); goat anti-rabbit IgG (H+L) (A11034); goat anti-rat IgG (H+L) (A11006); Alexa Fluor 555 goat anti-rabbit IgG (H+L) (A21429); goat anti-human IgG (H+L) (A21433); Alexa Fluor 647 goat anti-human IgG (H+L) (A21445); donkey anti-mouse IgG (H+L) (A31571) (1:500, Molecular Probes)
Validation	All commercial antibodies were validated by the manufacturers. Rabbit anti-Ndc80 antiserum was validated in Diaz-Rodríguez E. et al., 2008. Rabbit anti-CENP-C antibody was validated in Ishiguro et al., 2011. Human anti-centromere antibodies (CS1058), mouse anti-α-tubulin antibody (DM1A T6199), rabbit anti-Aurora A (NB100-2371), sheep anti-BubR1 (ab28192) and mouse anti-pericentrin (611814) were validated in Yoshida S et al. 2015. Rat anti-GFP antibody (GF090R 04404-84) was validated by the manufacture (https://www.nacalai.co.jp/ss/ec/EC-srchdetl.cfm?HP=1&l=EN&lc=1&syohin=0440484&syubetsu=3&catalog=&SiireC=&MakerC=&yoro=). Rabbit anti-Prc1 (sc-8356) was validated in Hu CK et al., 2012. Mouse anti-Ndc80 (9G3.23) was validated by the manufacture (https://www.genetex.com/Product/Detail/Hec1-antibody-9G3-23/GTX70268). Rabbit anti-HURP (sc-98809) was validated in Breuer M et al., 2010. Mouse anti-Mad2 (sc-65492) was validated in Kyogoku H and Kitajima T 2017. Mouse anti-GFP (ab1218) was validated by the manufacture (https://

www.abcam.co.jp/gfp-antibody-9f9f9-ab1218.html). Rat anti-α-tubulin (MCA77G) was validated in Courtois A et al., 2012. Rabbit anti-Kif11 (HPA010568) was validated in Clift D. et al., 2017. Mouse anti-Scp3 (ab97672) was validated in Jiang H. et al., 2018. Goat anti-cyclin B2(AF6004) was validated in Gui L. & Homer H. 2012. Rabbit anti-actin (ab1801) was validated by the manufacture (https://www.abcam.co.jp/actin-antibody-loading-control-ab1801.html). Alexa Fluor 488 goat anti-mouse IgG (H+L) (A11029); goat anti-rabbit IgG (H+L) (A11034); goat anti-rat IgG (H+L) (A11006); Alexa Fluor 555 goat anti-rabbit IgG (H+L) (A21429); goat anti-human IgG (H+L) (A21433); Alexa Fluor 647 goat anti-human IgG (H+L) (A21445); donkey anti-mouse IgG (H+L) (A31571) were validated by the manufacture (https://www.thermofisher.com/antibody/product/Goat-anti-Mouse-IgG-H-L-Highly-Cross-Adsorbed-Secondary-Antibody-Polyclonal/A-11029, https://www.thermofisher.com/antibody/product/Goat-anti-Rabbit-IgG-H-L-Highly-Cross-Adsorbed-Secondary-Antibody-Polyclonal/A-11034, https://www.thermofisher.com/antibody/product/Goat-anti-Rat-IgG-H-L-Cross-Adsorbed-Secondary-Antibody-Polyclonal/A-11006, https://www.thermofisher.com/antibody/product/Goat-anti-Rabbit-IgG-H-L-Highly-Cross-Adsorbed-Secondary-Antibody-Polyclonal/A-21429, https://www.thermofisher.com/antibody/product/Goat-anti-Human-IgG-H-L-Cross-Adsorbed-Secondary-Antibody-Polyclonal/A-21433, https://www.thermofisher.com/antibody/product/Goat-anti-Human-IgG-H-L-Cross-Adsorbed-Secondary-Antibody-Polyclonal/A-21445, https://www.thermofisher.com/antibody/product/Donkey-anti-Mouse-IgG-H-L-Highly-Cross-Adsorbed-Secondary-Antibody-Polyclonal/A-31571).

Diaz-Rodríguez E., Sotillo R., Schvartzman J.M., Benezra R. Hec1 overexpression hyperactivates the mitotic checkpoint and induces tumor formation in vivo. Proc. Natl. Acad. Sci. USA. 105, 16719–16724 (2008).
Ishiguro, K., Kim, J., Fujiyama-Nakamura, S., Kato, S. & Watanabe, Y. A new meiosis-specific cohesin complex implicated in the cohesin code for homologous pairing. Embo Rep 12, 267–275 (2011).
Yoshida, S., Kaido, M. & Kitajima, T. Inherent Instability of Correct Kinetochore-Microtubule Attachments during Meiosis I in Oocytes. Dev Cell 33, 589–602 (2015).
Hu, C.-K., Özlü, N., Coughlin, M., Steen, J. & Mitchison, T. Plk1 negatively regulates PRC1 to prevent premature midzone formation before cytokinesis. Mol Biol Cell 23, 2702–2711 (2012).
Breuer, M. et al. HURP permits MTOC sorting for robust meiotic spindle bipolarity, similar to extra centrosome clustering in cancer cells. The Journal of cell biology 191, 1251–60 (2010).
Kyogoku, H. & Kitajima T. Large Cytoplasm Is Linked to the Error-Prone Nature of Oocytes. Dev Cell 41(3):287-298 (2017).
Courtois A., Schuh M., Ellenberg J., Hiiragi T. The transition from meiotic to mitotic spindle assembly is gradual during early mammalian development. J Cell Biol. 2012 198(3):357-70.
Clift D., McEwan WA., Labzin LI., Konieczny V., Mogessie B., James LC., Schuh M.. A Method for the Acute and Rapid Degradation of Endogenous Proteins. Cell. 171(7):1692-1706 (2017).
Jiang H., Gao Q., Zheng W., Yin S., Wang L., Zhong L., Ali A., Khan T., Hao Q., Fang H., Sun X., Xu P., Pandita TK., Jiang X., Shi Q. MOF influences meiotic expansion of H2AX phosphorylation and spermatogenesis in mice. PLoS Genet. 24;14(5) (2018).
Gui, L. & Homer, H. Hec1-Dependent Cyclin B2 Stabilization Regulates the G2-M Transition and Early Prometaphase in Mouse Oocytes. Developmental Cell 25, 43–54 (2013).

Animals and other organisms

Policy information about studies involving animals; ARRIVE guidelines recommended for reporting animal research

Laboratory animals	B6D2F1 (C57BL/6 × DBA/2), Ndc80f/f and Ndc80f/f Zp3-Cre (C57BL/6 background) female mice, 8–12 weeks of age, were used to obtain oocytes. B6D2F1 males, 8–12 weeks of age, were used to obtain spermatocytes.
Wild animals	This study did not involve wild animals.
Field-collected samples	This study did not involve samples collected from the field.
Ethics oversight	All animal experiments were approved by the Institutional Animal Care and Use Committee at RIKEN Kobe Branch (IACUC).

Note that full information on the approval of the study protocol must also be provided in the manuscript.

Human research participants

Policy information about studies involving human research participants

Population characteristics	In the experiments in Fig. 7a, volunteer donors were women at 21, 24, 29 years old, with no history of infertility.<br>In the experiments in Fig. 7b and Supplementary Fig. 11c, volunteer donors were women under fertility treatment.
Recruitment	For the experiments in Fig. 7a, volunteer donors donated oocytes specifically for research, following informed consent.<br>For the experiments in Fig. 7b and Supplementary Fig. 11c, patients under fertility treatment donated oocytes after they received full explanation of the experiments and provided signed informed consent.
Ethics oversight	The experiments in Fig. 7a were approved by the Health Research Authority (UK), NRES Committee North East, Newcastle and North Tyneside1 Local Research Ethics Committee (REC reference 16/NE/0003). Vitrified oocytes were stored under a research license (R0152) from the UK Human and Fertilization Authority (HFEA).<br>The experiments in Fig. 7b and Supplementary Fig. 11c were approved by institutional human research ethics committees at RIKEN (KOBE-IRB-13-22) and IVF Namba Clinic (2014-1), registered in Japan Society of Obstetrics and Gynecology (registry number 132), and carried out under these guidelines.

Note that full information on the approval of the study protocol must also be provided in the manuscript.
